# Fever of Unknown Origin as an Isolated Manifestation of Ulcerative Colitis Despite Clinical and Radiographic Remission: A Case Report

**DOI:** 10.7759/cureus.110918

**Published:** 2026-06-15

**Authors:** Muhammad Memon

**Affiliations:** 1 Internal Medicine, ChristianaCare Health System, Newark, USA

**Keywords:** fever of unkown, hiv and aids, hyper inflamm, immuno suppresion, ulcerative colitis (uc)

## Abstract

Fever is a recognized manifestation of active ulcerative colitis (UC); however, isolated fever in the absence of GI symptoms or objective evidence of active colonic inflammation is exceedingly uncommon. The diagnostic challenge is amplified in immunocompromised patients receiving biologic therapy, in whom occult infection must be rigorously excluded. We present a 68-year-old woman with a history of well-controlled HIV infection and recently diagnosed UC who was initiated on vedolizumab, mesalamine, and a prednisone taper in the outpatient setting. She presented with persistent daily fevers and initial concern for an infectious etiology of pyrexia. Ulcerative colitis appeared to be well treated, with outpatient CT demonstrating resolution of colitis, and the patient denied diarrhea, abdominal pain, hematochezia, or other symptoms suggestive of active disease. An extensive infectious workup, including blood cultures, viral studies, fungal biomarkers, Lyme disease testing, tick-borne testing, and autoimmune serologies, was negative. During the laboratory evaluation, thrombocytopenia, markedly elevated inflammatory markers, and elevated D-dimer levels were noted. Despite a comprehensive multidisciplinary evaluation, no infectious, malignant, thrombotic, or rheumatologic etiology was identified. Throughout her hospital course, the patient developed sequelae of a severe inflammatory response, including atrial fibrillation. The patient's fevers were ultimately attributed to an atypical systemic inflammatory manifestation of UC. This case highlights a rare presentation of UC manifesting predominantly as fever of unknown origin (FUO) despite apparent clinical and radiographic remission. Clinicians should recognize that significant systemic inflammation may occur even in the absence of overt GI disease activity after exclusion of alternative causes.

## Introduction

Ulcerative colitis (UC) is a chronic inflammatory bowel disease characterized by relapsing and remitting mucosal inflammation of the colon. Clinical manifestations typically include diarrhea, hematochezia, abdominal pain, urgency, and tenesmus, although systemic symptoms such as fatigue, weight loss, and fever may occur in more severe disease activity [1]. In addition to intestinal symptoms, UC may be associated with a variety of extraintestinal manifestations and systemic inflammatory responses that contribute substantially to disease morbidity [2]. Fever is a recognized feature of active UC and has been reported in up to 40% of patients at presentation; however, it is generally accompanied by objective evidence of active intestinal inflammation and gastrointestinal symptomatology [3].

Fever of unknown origin (FUO) in patients with UC presents a significant diagnostic challenge, particularly among individuals receiving immunosuppressive or biologic therapies. The differential diagnosis is broad and includes opportunistic infections, malignancy, thromboembolic disease, medication-related adverse effects, and rheumatologic disorders. In patients with concomitant immunocompromising conditions such as HIV infection, infectious etiologies must be rigorously excluded before attributing fever to inflammatory bowel disease activity.

Vedolizumab is a monoclonal antibody targeting the α4β7 integrin and exerts a gut-selective anti-inflammatory effect by inhibiting lymphocyte trafficking to the gastrointestinal tract [4]. Although vedolizumab is associated with a favorable safety profile and a relatively low risk of systemic opportunistic infections compared with broader immunosuppressive therapies, persistent fever in patients receiving biologic treatment frequently prompts extensive evaluation for occult infection or other serious pathology [5-6].

While systemic inflammatory manifestations are well described in active UC, isolated fever occurring in the apparent absence of gastrointestinal symptoms or radiographic evidence of active colitis is exceedingly uncommon. Several case reports have described UC presenting as FUO; however, most cases ultimately demonstrate active intestinal disease upon further evaluation [3,7]. We describe a case of persistent daily fevers in a 68-year-old woman with well-controlled HIV infection and recently diagnosed UC undergoing treatment with vedolizumab, mesalamine, and a corticosteroid taper. Despite apparent clinical and radiographic remission of colitis and an exhaustive multidisciplinary evaluation, no infectious, malignant, thrombotic, or rheumatologic etiology was identified. The patient's fever was ultimately attributed to an atypical systemic inflammatory manifestation of UC, highlighting an uncommon presentation that clinicians should consider when evaluating FUO in patients with inflammatory bowel disease after alternative causes have been excluded.

## Case presentation

A 68-year-old woman with a past medical history significant for well-controlled HIV infection, recently diagnosed UC, prior diverticulitis, and chronic insomnia presented with persistent daily fevers and malaise. Her UC had recently been treated with vedolizumab (Entyvio), mesalamine, and a prednisone taper. Prior to presentation, her GI symptoms had improved substantially.

Computed tomography of the abdomen and pelvis performed prior to admission demonstrated resolution of previously identified colitis without evidence of active bowel inflammation, abscess, perforation, or other intra-abdominal pathology. This was repeated on admission and did not show active colitis (Figure 1).

**Figure 1:**
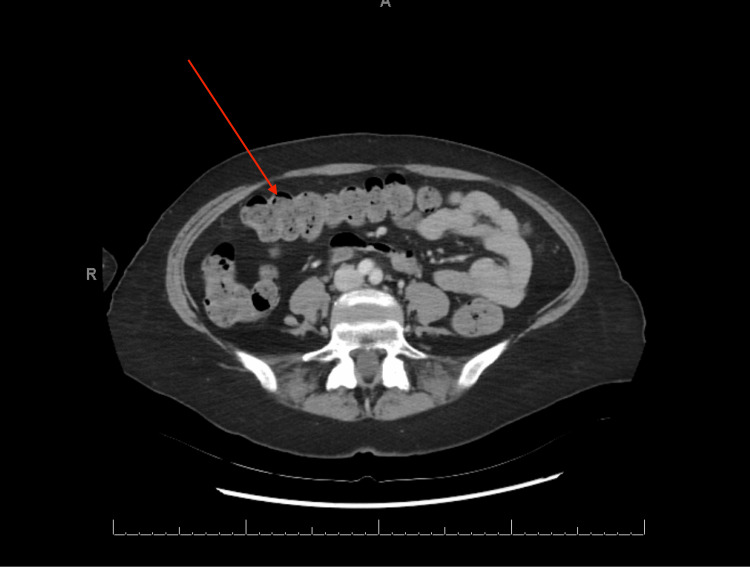
CT of the abdomen and pelvis with IV contrast in an axial/transverse view showing the colon without CT evidence of active inflammation.

At presentation, the patient denied abdominal pain, diarrhea, hematochezia, urgency, or other symptoms suggestive of an active UC flare. She denied nausea, vomiting, chills, shortness of breath, arthralgia, or any new rashes.

Her presenting chemistry laboratory values were largely unremarkable other than hyponatremia at 129. Other than thrombocytopenia, her initial complete blood count (CBC), including differential, was unremarkable, showing no neutropenia, eosinophilia, or other abnormalities. This is shown in Table 1.

**Table 1:** Initial notable laboratory findings on admission.

Laboratory test	Finding	Reference range
Sodium	129 mmol/L	136-145 mmol/L
Platelets	92 × 10³/µL	150-450 × 10³/µL

She developed a low-grade fever one day after admission at 38.2 degrees Celsius and then had daily high fevers with a maximum temperature of 39.4 degrees Celsius for 12 days after admission. This temperature trend is shown in Table 2.

**Table 2:** Daily trend of maximum temperature recorded throughout admission.

Day of admission	Maximum temperature (°C)	Reference range (°C)
1	38.2	36.1-38.0
2	39.1	36.1-38.0
3	39.4	36.1-38.0
4	39.1	36.1-38.0
5	38.5	36.1-38.0
6	38.2	36.1-38.0
8	38.2	36.1-38.0
10	39.2	36.1-38.0
11	38.2	36.1-38.0
12	38.4	36.1-38.0

Given her HIV status and immunosuppressive treatment regimen, an extensive infectious disease evaluation was pursued. Multiple blood cultures remained negative throughout hospitalization. Viral studies were unrevealing, including testing for Epstein-Barr virus (EBV), cytomegalovirus (CMV), and a comprehensive respiratory viral panel. Serum Fungitell and Aspergillus galactomannan assays were negative, arguing against invasive fungal infection. Lyme disease testing and a comprehensive tick-borne disease panel were negative. These findings are detailed in Table 3.

**Table 3:** Thorough evaluation of possible etiologies of fever. ^1^Cytomegalovirus ^2^Epstein-Barr virus ^3^Transthoracic echocardiogram ^4^Antinuclear antibody ^5^Anti-double-stranded DNA antibody ^6^Rheumatoid factor ^7^Hemophagocytic lymphohistiocytosis ^8^Ultrasound CMV: Cytomegalovirus; EBV: Epstein-Barr virus; IgG: Immunoglobulin G; IgM: Immunoglobulin M; TTE: Transthoracic echocardiogram; ANA: Antinuclear antibody; Anti-dsDNA antibody: Anti-double-stranded DNA antibody; RF: Rheumatoid factor; HLH: Hemophagocytic lymphohistiocytosis.

Category	Evaluation	Result
Bacterial	Blood cultures, urinalysis, urine Legionella antigen, cat scratch disease testing	Negative
Viral	CMV¹, EBV², respiratory viral panel	Negative
Fungal	Cryptococcal antigen, Fungitell, galactomannan, Histoplasma urine antigen	Negative
Parasitic	Toxoplasma antibodies, IgG and IgM	Negative
Tick-borne illness	Lyme titer, Babesia PCR, Ehrlichia testing, Anaplasma testing	Negative
Endocarditis	Blood cultures, TTE³	Negative
Malignancy	CT of the chest, abdomen, and pelvis	Negative
Autoimmune	ANA⁴, anti-dsDNA antibody⁵, RF⁶	Negative
Hyperinflammatory	Still's disease and HLH⁷ evaluation	Negative
Advanced pathogen testing	Karius testing	Negative
Clot	US⁸ of the lower extremities	Negative
Medication	Medication review	Negative

Computed tomography of the chest, abdomen, and pelvis failed to identify an infectious source, occult abscess, lymphadenopathy, splenomegaly, malignancy, or evidence of recurrent colitis. Given persistent fever despite a negative conventional infectious evaluation, plasma microbial cell-free DNA testing (Karius assay) was obtained and did not identify a clinically significant pathogen. 

The patient also underwent evaluation for subacute infective endocarditis. Serial blood cultures remained negative, and transthoracic echocardiography demonstrated no valvular vegetations or other findings suggestive of endocarditis. Deep vein thrombosis (DVT) was also considered; however, ultrasound studies were negative. These findings are noted in Table 3.

Because HIV infection is associated with an increased risk of lymphoma and other malignancies that may present with FUO, malignancy was carefully considered. However, CT imaging of the chest, abdomen, and pelvis demonstrated no evidence of lymphadenopathy, mass lesions, hepatosplenomegaly, or other findings concerning for lymphoma or occult malignancy. These findings are also mentioned in Table 3.

Further laboratory evaluation was notable for thrombocytopenia and elevated inflammatory markers, including an elevated D-dimer. Given the persistent unexplained fevers and elevated inflammatory markers, ferritin and triglyceride levels were obtained to evaluate for Still's disease and hemophagocytic lymphohistiocytosis (HLH). Her CD4 count was transiently decreased compared with baseline, likely reflecting recent corticosteroid and biologic therapy. These laboratory values are shown in Table 4.

**Table 4:** Abnormal laboratory values. ^1^Erythrocyte sedimentation rate ^2^C-reactive protein

Laboratory test	Finding	Reference range
D-dimer	8,121 ng/mL	<244 ng/mL
ESR¹	23 mm/hr	0-20 mm/hr
CRP²	183.7 mg/L	1-5 mg/L
Ferritin	607 ng/mL	5-204 ng/mL
Absolute CD4 count	86 cells/µL	404-1,612 cells/µL

Given the combination of persistent fever, elevated inflammatory markers, thrombocytopenia, and elevated ferritin, rheumatologic and hematologic evaluations were pursued. A broad autoimmune workup was negative. This is noted in Table 3.

The patient was started on empiric broad-spectrum antibiotics with ceftriaxone and doxycycline due to unremitting fevers. Her fevers improved after 1 day of antibiotic treatment; however, they returned as daily fevers. After multiple days of antibiotics without improvement, the empiric antibiotics were discontinued.

Approximately two weeks into hospitalization, the patient developed new-onset atrial fibrillation with rapid ventricular response requiring rapid response team activation and escalation of care. The arrhythmia was successfully managed medically.

Despite extensive multidisciplinary evaluation involving infectious disease, hematology, rheumatology, cardiology, and gastroenterology services, no infectious, malignant, autoimmune, or thromboembolic etiology for the patient's persistent fevers was identified.

Drug-induced fever was considered; however, all drugs that the patient was started on at admission had been previously tolerated prior to the fever. Antibiotic-induced fever was considered unlikely, as the first evidence of fever occurred before antibiotics were administered. Additionally, her CBC did not show any evidence of eosinophilia.

After 15 days of hospitalization and multiple negative findings, fevers related to UC were considered, and a fecal calprotectin level was obtained and returned elevated at 498 mcg/g, as shown in Table 5.

**Table 5:** Patient's elevated fecal calprotectin level.

Evaluation	Patient value	Reference range
Fecal calprotectin	498 µg/g	<50 µg/g

Given the recent diagnosis of UC, elevated inflammatory markers, and exhaustive negative workup, the patient's fevers were ultimately attributed to an atypical systemic inflammatory manifestation of UC despite radiographic resolution of colitis and absence of gastrointestinal symptoms, as a diagnosis of exclusion. Endoscopic evaluation and biopsy were not performed due to consideration of the patient's clinical status.

## Discussion

Fever is a recognized systemic manifestation of UC and is most commonly encountered during periods of active intestinal inflammation. Patients typically present with diarrhea, hematochezia, abdominal pain, urgency, and constitutional symptoms that correlate with the degree of colonic disease activity [1]. Although fever may accompany moderate-to-severe disease, its occurrence as the predominant or isolated manifestation of UC is distinctly uncommon [3]. The absence of gastrointestinal symptoms in our patient substantially broadened the differential diagnosis and necessitated an extensive evaluation for alternative causes of FUO.

Several reports have described UC presenting initially as FUO. One report described a patient whose primary manifestation of previously undiagnosed UC was persistent fever, highlighting inflammatory bowel disease as an important noninfectious consideration in the evaluation of FUO after exclusion of infectious etiologies [3]. Similarly, another report described an elderly patient who underwent a prolonged diagnostic workup for unexplained fever before active UC was ultimately identified as the underlying cause [7]. Notably, these previously reported cases ultimately demonstrated active intestinal inflammation upon further evaluation. In contrast, our patient had recently diagnosed UC that appeared to be in clinical and radiographic remission, making attribution of her fever to UC considerably more challenging.

The diagnostic complexity of this case was further amplified by the patient's well-controlled HIV infection and recent initiation of biologic therapy. In immunocompromised individuals, persistent fever warrants a thorough investigation for opportunistic infection, medication-related complications, malignancy, and thromboembolic disease. Although vedolizumab has a favorable safety profile and is associated with lower rates of systemic immunosuppression compared with many other biologic agents, concerns regarding occult infection remain paramount in patients presenting with unexplained fever while receiving biologic therapy [4-5]. Accordingly, our patient underwent an extensive infectious, hematologic, rheumatologic, and thrombotic evaluation, all of which proved unrevealing.

The most striking feature of this case is the apparent dissociation between systemic inflammation and intestinal disease activity. Cross-sectional imaging performed prior to admission and during hospitalization demonstrated resolution of previously documented colitis, and the patient denied diarrhea, abdominal pain, hematochezia, urgency, or other symptoms suggestive of active disease. Nevertheless, she exhibited persistent daily fevers, markedly elevated inflammatory markers, thrombocytopenia, and elevated D-dimer levels, indicating a robust systemic inflammatory response. Her hospitalization was further complicated by atrial fibrillation with rapid ventricular response, a condition increasingly recognized to occur in the setting of significant systemic inflammation.

Although the precise mechanism underlying this presentation remains uncertain, UC is increasingly understood as a systemic immune-mediated disorder rather than a disease confined exclusively to the colonic mucosa. Dysregulated immune activation and cytokine production, including tumor necrosis factor-α, interleukin-1, interleukin-6, and interleukin-17, contribute not only to intestinal injury but also to extraintestinal and constitutional manifestations [3]. Persistent immune activation has been demonstrated in some patients despite clinical remission, suggesting that symptom resolution and even radiographic improvement may not necessarily reflect complete immunologic quiescence [3]. Consequently, systemic inflammatory manifestations may occasionally predominate even when overt GI disease activity is absent.

This case expands the limited literature describing UC as a cause of FUO and highlights a particularly unusual presentation in which persistent fever occurred despite apparent clinical and radiographic remission of colitis. After exclusion of infectious, malignant, thrombotic, and rheumatologic etiologies, UC-related systemic inflammation emerged as the most plausible diagnosis of exclusion for the patient's presentation. Clinicians should remain aware that significant inflammatory activity may rarely occur independent of gastrointestinal symptoms, particularly when evaluating persistent unexplained fever in patients with inflammatory bowel disease. Recognition of this atypical manifestation may facilitate earlier diagnosis and avoid unnecessary diagnostic testing once alternative causes have been thoroughly excluded.

## Conclusions

FUO may represent a rare extraintestinal manifestation of UC, even in the absence of GI symptoms and despite radiographic resolution of colonic inflammation. In patients undergoing exhaustive evaluation with persistently elevated inflammatory markers and exclusion of infectious, malignant, and autoimmune causes, UC-associated systemic inflammation should remain a diagnostic consideration. If feasible, endoscopic evaluation should be completed with biopsies to assess for active disease. Recognition of this atypical presentation may prevent delays in diagnosis and management.
